# Influence of lysosomal sequestration on multidrug resistance in cancer cells

**DOI:** 10.20517/cdr.2018.23

**Published:** 2019-03-19

**Authors:** Reginald Halaby

**Affiliations:** Department of Biology, Montclair State University, Montclair, NJ 07043, USA.

**Keywords:** Lysosomal sequestration, lysosomes, chemotherapeutics, multidrug resistance, cancer, permeability-glycoprotein, exocytosis

## Abstract

Chemotherapy remains a primary treatment modality for various malignancies. However, resistance to chemotherapeutic drugs is a major obstacle to curative cancer therapy. Lysosomes are acidic organelles that participate in cellular digestion. However, there is rising interest in lysosomes because of their involvement with cancer. For example, extracellular secretion of lysosomal enzymes promote tumorigenesis; cytosolic leakage of lysosomal hydrolases promote apoptosis; and weak chemotherapeutic bases diffuse across the lysosomal membrane and become entrapped in lysosomes in their cationic state. Lysosomal drug sequestration lowers the cytotoxic potential of chemotherapeutics, reduces drug availability to sites of action, and contributes to cancer resistance. This review examines various mechanisms of lysosomal drug sequestration and their consequences on cancer multidrug resistance. Strategies for overcoming drug resistance by exploiting lysosomes as subcellular targets to reverse drug sequestration and drug resistance are also discussed.

## Introduction

Lysosomes are acidic organelles that contain over 50 digestive enzymes that can degrade all macromolecules. They are the major cell digestive organelles^[1]^. In addition to this housekeeping function, lysosomes perform diverse cellular processes. For example, they are involved in macroautophagy, chaperone-mediated autophagy, cholesterol homeostasis, and degrading receptor tyrosine kinase receptors and growth factors^[2-6]^. Lysosomal hydrolases play opposing roles in neoplastic cells. Lysosomal proteases that are secreted extracellularly promote tumor invasion and metastasis^[7-9]^. Specifically, cathepsins are used by tumor cells to degrade extracellular matrix components such as fibronectin, elastin, and laminin, thereby facilitating invasion, angiogenesis, and metastasis^[10,11]^. In contrast, cytosolic translocation of lysosomal proteases induces apoptosis and cell death^[12,13]^. Due to the dual role that lysosomes have in cancer, mounting evidence suggests that they are attractive targets for oncological therapies^[14,15]^.

Drug resistance is the primary reason that cancer treatments fail and patients die. Multidrug resistance (MDR) occurs in cancer cells that develop the ability to resist various drugs that are structurally and pharmacologically unrelated^[16]^. It is well documented that the drug transporter permeability-glycoprotein (P-gp) contributes to MDR^[17]^. Al-Akra and his team demonstrated that lysosomal P-gp plays an important role in conferring drug resistance^[18]^. Additionally, P-gp has been observed to localize to lysosomes^[19]^. In spite of novel approaches to address MDR, it continues to be a major cause of unsuccessful cancer treatments^[20,21]^.

Mounting evidence shows that lysosomes play a role in MDR. A major limitation of chemotherapeutic drugs is that they become trapped or sequestered in acidic organelles, such as the lysosomes. Lipophilic chemotherapeutic agents with weak base properties readily diffuse across cell membranes. However, when these drugs enter the acidic lumen of lysosomes, they become protonated and are trapped in the lysosomes^[22]^. Mounting evidence has demonstrated that certain hydrophobic weak base chemotherapeutics such as the anthracyclines doxorubicin, daunorubicin, and mitoxantrone, imidazoacridinones, the receptor tyrosine kinase inhibitor sunitinib, and pyrimethamine preferentially accumulate in lysosomes^[23-30]^. Lysosomal sequestration markedly reduces drug concentrations and thereby directly decreases the effects of anticancer drugs on their intended targets, the nucleus and cytoplasm. This lysosomal scavenging of oncologic drugs potentiates MDR. Therefore, understanding the molecular mechanism underlying lysosomal sequestration of chemotherapeutic drugs should provide insights to circumvent this clinical problem.

## Lysosomal membrane permeabilization

Lysosomes can also initiate the intrinsic apoptosis pathway in response to treatment by lysosomotropic agents. Li *et al.*^[31]^ (2000) were one of the first groups to demonstrate that lysosomotropic agents disrupted the lysosomal membrane, resulting in cytosolic leakage of the acid hydrolases, specifically the cathepsins, and apoptosis. Subsequently, various studies have confirmed that lysosomal membrane permeabilization (LMP) induces apoptosis^[12,32,33]^.

Since LMP is known to trigger apoptosis in various cancer cells, drug screens to identify agents that induce LMP may prove to be effective cancer therapies to overcome drug resistance induced by lysosomal drug sequestration. For example, a recent study detected 175 compounds that induced death in HCT116 colon cancer cells. Notably, over half of the 11 compounds that induced apoptosis in p-53 deficient cells did so by LMP and cathepsin-mediated cell death^[34]^. Additionally, the hydrophobic weak base siramesine induced LMP in cancer cells *in vitro* and *in vivo*^[35,36]^. Consistent with this approach, bovine α-lactalbumin and oleic acid was shown to kill various cancer cell lines (L1210 leukemia, HeLa cervical adenocarcinoma, PC-3 prostate adenocarcinoma, U118 MG glioblastoma, MCF-7 breast adenocarcinoma and others) by a mechanism involving LMP^[37]^.

## Cancer cells modify lysosomes to evade cell death

The success of cancer cells to develop resistance to chemotherapeutics also involves mutating pro-apoptotic pathways and lysosomal-mediated death pathways while upregulating cell proliferation pathways. Tumor cells use various methods to modify their lysosomes in an effort to evade cell death. The increased activity of phosphatidylinositol-3’-kinase (PI3K), which is characteristic of many tumors^[38-40]^, promotes stability in tumor cell lysosomes. Mousavi *et al.*^[41]^ (2003) reported that PI3K regulates the size, maturation, and activity of lysosomes. Tumor cells can abolish LMP by overexpression of cytosolic protease inhibitors^[42,43]^. Cancer cells also protect themselves from LMP by translocating Hsp70 from the cytosol to the lysosomal lumen where it stabilizes lysosomal membranes by promoting the activity of acid sphingomyelinase^[44,45]^. Support for this role of Hsp70 comes from observations that depletion of this protein triggers a tumor-cell-specific lysosomal cell death program^[46]^.

## Lysosomal involvement in MDR

### Lysosomal sequestration of weak bases

Lysosomes have been shown to sequester lipophilic, weakly basic chemotherapeutic drugs via a non-enzymatic and non-transporter mediated mechanism^[23]^. Notably, adriamycin was observed to concentrate in the lysosomes of drug-resistant cells but not in lysosomes drug-sensitive cells^[47]^. Presumably these weak bases are freely transported to the lysosomes by passive transport due to their hydrophobic composition. The pKa values, predominantly above 7.0, for these drugs confirms that they are weak bases. Likewise, the intracellular efficacy of these compounds can be decreased *in vitro* at acidic pH^[23,25]^. Many of the drugs used to treat malignancies are weak bases, and it has been demonstrated by several reports that they become sequestered in lysosomes. These include the following drugs: daunorubicin, doxorubicin, lapatinib, vincristine, and nintedanib^[23,25,27,36,48]^. Once they cross the lysosomal membrane, these weak base chemotherapeutics become trapped through protonation in the lysosomal lumen^[49,50]^. The use of the term “drug trapping” in lysosomes can be misleading. The ionized form, in most cases, is in equilibrium with the neutral drug in the cytosol. Furthermore, the ionized version of the drug can rapidly cross the lysosomal membrane by passive diffusion when the cytosolic concentration of the drug decreases due to transport into the bile, metabolism, or diffusion back into the plasma^[23]^. Support for this comes from the observation of rapid reversibility of lysosomal trapping of a lipophilic drug seen when rats are asphyxiated with carbon dioxide, which modestly acidifies the blood and causes a decrease in tissue levels and an increase in plasma drug levels^[51]^. These results suggest that lysosomes function as a reservoir pulling the drug from its target site and do not indefinitely trap cytotoxic drugs.

In contrast, localization of chemotherapeutics to the acidic lumen of lysosomes does promote MDR. Support for this comes from several lines of evidence. Lysosomal accumulation of sunitinib was detected in hepatocellular carcinoma cells, renal cancer cells, and colon cancer cells^[24,52]^. The multikinase inhibitor nintedanib has been investigated in clinical trials for the following tumors: non-small cell lung cancer, colorectal cancer, prostate cancer, and pancreatic cancer^[53-56]^. One study reported that nintedanib was sequestered in lysosomes, thus lowering its cytosolic concentrations and its fibroblast growth factor receptor inhibition potential^[48]^. Another report found that intracellular levels of imatinib are primarily determined by lysosomal sequestration^[57]^. Lysosomes have also been reported to have indirect effects on drug sequestration. Kalayda *et al.*^[58]^ reported that abnormalities in the lysosomal compartment promote sequestration of cisplatin away from the nucleus due to faulty localization of transport proteins. Taken together, these data suggest that anticancer drug resistance is modulated by lysosomal sequestration, as summarized in Table 1. Further studies are warranted to determine whether targeting lysosomes can overcome resistance to chemotherapeutics.

**Table 1:** Chemotherapeutics that are sequestered in lysosomes and confer drug resistance

Drug	Molecular target	References
Doxorubicin	Topoisomerase II inhibitor	[25]
Vinblastine	Antimicrotubule agent	[68]
Vincristine	Antimicrotubule agent	[70]
Methotrexate	Dihydrofolate reductase	[29]
Sunitinib	VEGFR2, PDGFRb, c-kit	[24]
Pyrimethamine	Dihydrofolate reductase	[30]
Lapatinib	EGFR, HER2	[23]
Gefitinib	EGFR	[23]
Sorafenib	RAF, VEGFR	[52]
Nintedanib	VEGFR, FGFR, PDGFR	[48]
Topotecan	Topoisomerase I inhibitor	[83]
Imatinib	BCR-ABL	[48]
Pazopanib	VEGFR, PDGFR	[64]
Erlotinib	EGFR	[64]

VEGFR: vascular endothelial growth factor receptor; PDGFRb: beta-type platelet-derived growth factor receptor; EGFR: endothelial growth factor receptor; HER: human epidermal growth factor receptor; FGFR: fibroblast growth factor receptor; RAF: rapidly accelerated fibrosarcoma

Chemotherapeutic drugs are distributed between cytosolic and nuclear compartments intracellularly. Since lysosomes are not the intended target sites of these drugs, their entrapment in lysosomes effectively decreases their therapeutic effects at the wild type targets, such as nuclear DNA. Support for this notion comes from a study that showed that daunorubicin accumulation in lysosomes resulted in decreased nuclear concentrations of daunorubicin and drug resistance^[25]^. Likewise, another study found that lysosomal sequestration of doxorubicin in MCF-7/adriamycin breast cancer cells decreased the levels of the drug in the nucleus^[47]^. Indeed, it was reported that cells with a higher number of lysosomes were more resistant to sunitinib compared to cells with lower numbers of lysosomes^[49]^. Conflicting evidence comes from a study showing a cisplatin-resistant ovarian cancer cell line containing considerably fewer lysosomes than wild-type cells^[59]^.

### Lysosomal biogenesis and MDR

Lysosomal biogenesis is typically a response to cell stress and is regulated by the translocation of transcription factor EB (TFEB) from the cytosol to the nucleus^[60]^. TFEB activity appears to be regulated through its phosphorylation by mammalian target of rapamycin complex 1 (mTORC1). mTORC1 was shown to exert its kinase activity on lysosomal surfaces where it phosphorylates TFEB, thereby inactivating the transcription factor^[61]^. TFEB can be dephosphorylated by calcineurin, which transforms it to its active form and facilitates its nuclear translocation^[62]^. TFEB-mediated lysosomal biogenesis is induced by various stimuli, namely cell starvation, inhibition of mTORC1, and abnormal lysosomal storage^[60]^. Interestingly, lysosomal stress also modulates lysosomal gene expression^[63]^. A recent report showed that doxorubicin and mitoxantrone triggers TFEB-associated lysosomal biogenesis, thus further enhancing lysosomal sunitinib entrapment and MDR^[49]^. A different study found that exposure of 786-O renal cancer cells and HT-29 colorectal cancer to various tyrosine kinase inhibitors increased the number of lysosomes^[64]^. Taken together, these data lead credence to the notion that lysosomal drug sequestration induces lysosomal stress and TFEB-mediated lysosomal biogenesis. The number of lysosomes in cancerous cells may prove to be an important consideration when selecting treatment options for cancer patients. A possible solution may be to target lysosomal biogenesis by finding ways to circumvent the nuclear translocation of TFEB. Support for this comes from a study that found that interaction of TFEB with active rag heterodimers promoted recruitment of TFEB to lysosomes, leading to mTORC1-dependent phosphorylation and inhibition of TFEB^[65]^.

### Lysosomal sequestration mediated by ATP-binding cassette transporter proteins

**Figure 1:**
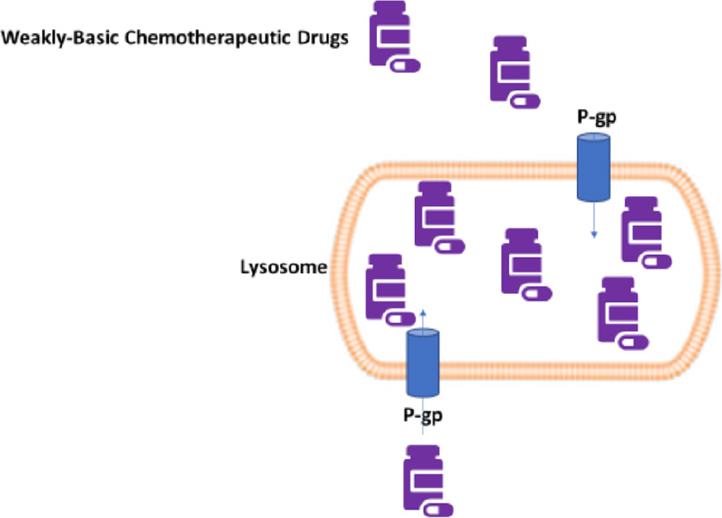
Lysosomal drug sequestration (LDS). P-gp expressed on lysosomal membranes contributes effluxes weakly-basic chemotherapeutics from the cytosol into the lysosomal lumen. LDS will decrease the cytosolic concentration of the drugs and their availability to molecular targets. P-gp: permeability-glycoprotein

Another mechanism by which lysosomes participate in drug sequestration involves ATP-binding cassette transporters (ABC-transporters; See Figure 1).

P-gp is often a representative ABC-transporter, present in many malignant cells and a molecular target in cancer therapies^[66,67]^. P-gp is located on cell membranes. However, since P-gp expression also exists on the lysosomal membrane, lysosomal P-gp can transport cytotoxic agents into lysosomes. Support for this notion comes from reports that chemotherapeutics that are P-gp substrates and ionize at lysosomal pH (pH 5), such as doxorubicin, danorubicin, vinblastine, and imatinib become localized and trapped inside lysosomes^[57,68]^. The pH difference between the cytosol and lysosomal lumen is the driving force for lysosomal drug sequestration and is regulated by proton-pumping vacuolar-ATPase proteins^[69]^. This mechanism prevents such drugs from reaching their pharmacologic cytosolic concentrations and contribute to survival of the tumor cells. Lysosomal P-gp-mediated MDR can be overcome by using specific P-gp inhibitors or a combination of lysosomotropic agents with anticancer drugs. Support for this comes from work by Shiraishi *et al.*^[70]^ (1986) demonstrating that chloroquine partially reversed the resistance of multi-drug-resistant KB carcinoma cells to the P-gp substrates adriamycin, daunomycin, vincristine, vinblastine and actinomycin D. Furthermore, it has been shown that P-gp inhibitors valspodar and elacridar or silencing P-gp with siRNA reversed lysosomal sequestration of doxorubicin, leading to its redistribution to its intended target, the nucleus^[68]^. A recent report demonstrated that cell-surface P-gp is degraded by the lysosomal pathway and suggests that this pathway could be exploited to induce cell death in P-gp expressing tumors^[71]^. Lastly, lysosomal P-gp mediated resistance to sorafenib was reversed in hepatocellular carcinoma cells that were incubated with verapamil after drug pre-incubation^[52]^. However, the majority of clinical trials using P-gp inhibitors to suppress drug resistance have failed to show improved survival or remission rates^[72]^.

Other transporter proteins have also been implicated in promoting lysosomal drug sequestration. The ABC transporter A3 (ABCA3) was shown to contribute to lysosomal sequestration of imatinib and to potentiate resistance to this drug^[73]^. Indeed, the majority of the intracellular concentration of imatinib was found not in the cytosol, rather it was localized to lysosomes^[73]^. An additional obstacle is the fact that ABCA3 mediated resistance is correlated with an increase in lysosomal-related organelles^[73]^.

### Lysosomal exocytosis potentiates MDR

Lysosomal exocytosis promotes MDR in malignant cells. Lysosomal exocytosis is a Ca^2+^-dependent process whereby lysosomes fuse with the plasma membrane and release their contents to the extracellular space^[74]^. Interestingly, lysosomal exocytosis is also regulated by TFEB and overexpression of TFEB is correlated with increased exocytosis^[75]^. It has been hypothesized that exocytosis of lysosomal sequestered drugs is another mechanism that contributes to reducing the concentration and efficacy of these drugs. Support for this comes from a study that found lysosomal exocytosis triggered in murine macrophages by treatment with agents that induced lysosomal alkalization^[76]^. These data lead credence to the notion that drugs that are sequestered in lysosomes are not trapped there indefinitely and are extruded via lysosomal exocytosis. Furthermore, lysosomal exocytosis of cathepsins B, D, K, and L have been shown to promote cell motility, angiogenesis, and metastasis^[7,77]^. Cathepsin D stimulates mitogen activated protein kinase signaling and angiogenic gene expression^[77]^.

## Approaches to reverse lysosomal sequestration

### Alkalinizing agents

**Figure 2:**
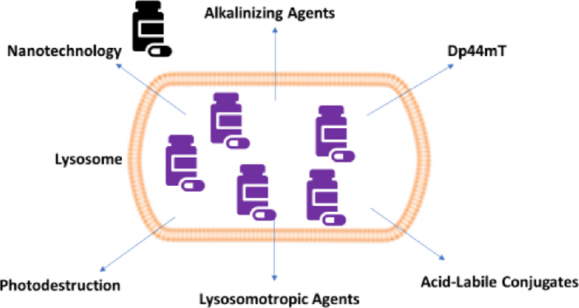
Putative strategies that may reverse lysosomal drug sequestration. Alkalinizing agents, nanotechnology, Dp44mT, photodestruction, lysosomotropic agents, and acid-labile conjugates may be employed to bypass lysosomal-mediated drug resistance. The purple medicine bottles represent chemotherapeutic drugs that are trapped in the lysosomal lumen and the black medicine bottle represents drugs that presumably will be sent back into the cytosol. Dp44mT: di-2-pyridylketone 4,4-dimethyl-3-thiosemicarbazone

We hypothesize that several mechanisms may reverse lysosomal drug sequestration. In the section below, we discuss putative strategies that might enable drugs that are trapped in lysosomes to translocate from lysosomal lumen to cytosol [Figure 2]. This would presumably aid in increasing drug availability to target sites.

Disrupting the acidification of lysosomes in multidrug-resistant cells has been shown to sensitize them to chemotherapeutics. A possible mechanism to reverse lysosomal drug accumulation of weak chemotherapeutic bases is by treatment with lysosome alkalinizing agents such as bafilomycin A1, a vesicular H^+^-ATPase inhibitor^[78]^. Bafilomycin A1, however, is too toxic for *in vivo* use and a more appropriate alkalinizing agent is chloroquine. One study administered chloroquine to mice and inhibited lysosomal function by raising the lysosomal pH^[79]^. A different study found that chloroquine potentiates the cytotoxic effects of doxorubicin in liver carcinoma cells^[80]^. Similarly, treatment of lysosomes in resistant cells with monensin, bafilomycin A1, or concanamycin A was sufficient to change the distribution of adriamycin to mimic that of drug-sensitive cells^[47]^. Additionally, prevention of subcellular trapping of nintedanib by lysosomal alkalinization abolished drug resistance^[48]^. These approaches appear plausible because pH gradient differences exist between MDR cancer cells and their wild-type drug sensitive counterpart cell lines^[81]^. These findings suggest that the use of well-tolerated alkalinizing agents may circumvent lysosomal drug sequestration and thereby increase cytotoxic drug efficacy.

### Lysosomotropic agents

Another approach to abolish lysosomal drug sequestration is by using lipophilic drugs that become scavenged in lysosomes yet can induce LMP^[70]^. Chloroquine has been reported to promote cytotoxicity and to act synergistically with chemotherapeutic drugs. Chloroquine is a lysosomotropic agent that triggers destabilization of the lysosomal membrane in various tumor cells. In one study chloroquine was used to restore sensitivity to cisplatin in refractory non-small-cell lung cancer cells^[82]^. In another report, chloroquine was shown to potentiate the cytotoxic effects of topotecan by inhibiting autophagy^[83]^. The thiosemicarbazone, di-2-pyridylketone 4,4-dimethyl-3-thiosemicarbazone (Dp44mT), has been shown to accumulate in lysosomes of tumor cells where it induces LMP^[84]^. Once in the lysosome, Dp44mT binds to copper forming a complex capable of producing cytotoxic reactive oxygen species (ROS) which triggers LMP^[84]^.

### Use of conjugates

Conjugation of acid-labile chemicals to chemotherapeutic drugs has also been utilized to overcome lysosomal drug sequestration. Hydrazone is a commonly used linker molecule for this purpose because of its stability at cytosolic pH and its ability to hydrolyze at lysosomal pH^[85,86]^. Evidence for this comes from a report that conjugated doxorubicin to polyamidoamine dendrimers via hydrazone^[87]^. This complex was shown to release doxorubicin to the nucleus and induce cell death^[87]^. Additionally, degradable peptides, which are digested by lysosomal hydrolases, have been conjugated to cancer drugs and overcame lysosomal trapping^[88]^.

### Photodestruction

Lysosomal photodestruction of weakly basic chemotherapeutics that are also fluorochromes is another approach to reverse lysosomal sequestration. Photodestruction of imidazoacridinone-loaded lysosomes in MDR cancer cells resulted in cell lysis via formation of ROS^[26]^. Another study combined sunitinib with phototherapy to combat lysosomal localization of the drug^[89]^. However, this approach is of limited value because of the superficial and local treatment options of phototherapy.

### Dp44mT

A recent study investigated the effects of glucose availability in cancer cells on reversing Pg-induced lysosomal drug entrapment. Seebacher and co-workers demonstrated that the anti-tumor agent and P-gp substrate Dp44mT induces LMP rather than lysosomal sequestration in response to glucose-induced stress^[90]^. When tumor cells are exposed to cellular stress, they produce increased amounts of ROS resulting in upregulation of P-gp expression^[91]^. As a result, P-gp actively pumps Dp44mT into lysosomes where it binds to copper, thereby forming ROS and triggering lysosomal membrane destabilization and apoptosis^[92]^. Lysosomes in neoplastic cells presumably have higher concentrations of copper compared to normal cells due to their increased requirement for metals^[93]^. The higher levels of copper are presumably needed by tumors for angiogenesis and metastasis^[94,95]^. Moreover, P-gp inhibitors such as elacridar abolished LMP induced by Dp44mT^[92]^. These findings suggest that Dp44mT only uses P-gp and not other ABC transporter proteins to overcome lysosomal drug sequestration. Taken together these results indicate that the design of novel metal-binding, P-gp substrate drugs like Dp44mT may be used to treat multidrug resistant tumors by targeting lysosomes.

### Nanotechnology

Nanomedicine is another mechanism that shows promising results to overcome P-gp mediated MDR. A study showed that doxorubicin-loaded nanospheres (DOX-NS) evaded MDR and delivered a high concentration of the drug to the nucleus and cytosol^[96]^. This finding suggests that DOX-NS was not recognized as a P-gp substrate. Another study added a monoclonal antibody 2C5, which recognizes various tumor cells via tumor cell surface-bound nucleosomes, to doxorubicin liposomes^[97]^. The antibody-doxorubicin liposome complex significantly induced nuclear accumulation and cytotoxicity of doxorubicin in a doxorubicin-resistant colon cancer cell line^[97]^.

## Conclusion

After long-term treatment with weakly basic anticancer drugs, lysosomal drug sequestration can occur. Lysosomes are attractive subcellular targets for creation of novel anticancer treatments for the following reasons. Tumor cells have larger, more active lysosomes, which make them more susceptible to lysosomal membrane degradation compared to lysosomes in non-neoplastic cells^[98]^. Additionally, cancer cells display higher metabolic rates and turnover of iron-containing proteins that sensitize them to ROS-induced LMP^[99]^. A novel imaging technique that can stably track lysosomes for at least 120 h, irrespective of pH changes in the organelle, is now available and can be used to monitor lysosomes in cancer cells^[100]^. This new lysosomal tracing method is desirable over conventional acidotropic probes, which tend to dissipate in stressed lysosomes. The above characteristics of lysosomes in cancer cells should be fully exploited to trigger lysosomal-mediated cell death [Figure 1].

We have reviewed the important role played by lysosomes in MDR by three mechanisms: (1) lysosomal entrapment of weakly basic chemotherapeutics; (2) P-gp-mediated lysosomal sequestration; and (3) lysosomal exocytosis of anticancer drugs. Lysosomal potentiation of MDR is an issue that is compounded by the following factors. As described above, several of the weakly basic chemotherapeutic drugs are also P-gp substrates that are involved in P-gp-mediated lysosomal entrapment. Upregulated biogenesis of lysosomes in cancer cells leads to enlargement of the lysosomal compartment^[101]^. The enlarged compartment allows for a significant amount of drug to be scavenged from sites of action. Evidence for this comes from studies that demonstrated that drugs that localize to lysosomes can reach intracellular concentrations that are markedly higher than drug concentrations in the surrounding medium^[102,103]^. Similarly, lysosomal exocytosis of drugs reduces their intracellular concentrations and cytotoxic effects. There is a dire need to develop new strategies to overcome MDR in cancer treatment. Lysosomes, with acid hydrolases that can trigger the intrinsic apoptotic pathway and trigger caspase activation, serve as attractive targets for novel anticancer treatment modalities^[104]^. Specifically, the lysosomes of tumor cells exhibit alterations that are not observed in normal cells: increased cathepsin activity, shifts in different endolysosomal populations, and modified lysosomal trafficking^[14]^. Further studies are warranted to fully exploit the unique differences in cancer lysosomes compared to their normal cell counterparts to sensitize tumor cells to cell death. The results of such projects should provide more effective strategies to bypass lysosomal-mediated drug resistance.

## Declarations

### Copyright

© The Author(s) 2019.
